# Comparison of Material Properties of Multilayered Laminates Determined by Testing and Micromechanics

**DOI:** 10.3390/ma14040761

**Published:** 2021-02-05

**Authors:** Maciej Kulpa, Agnieszka Wiater, Mateusz Rajchel, Tomasz Siwowski

**Affiliations:** Department of Roads and Bridges, Rzeszow University of Technology, 35-959 Rzeszow, Poland; wiater@prz.edu.pl (A.W.); mrajchel@prz.edu.pl (M.R.); siwowski@prz.edu.pl (T.S.)

**Keywords:** FRP composites, material properties, tension test, shear test, compression test, micromechanics, bridge deck

## Abstract

This paper presents an experimental material campaign focusing on fiber-reinforced polymers (FRP) to be applied in a novel bridge deck panel. Laminas based on most commonly used fibers, i.e., glass, carbon, basalt and aramid, were prepared and studied in tension, shear and compression. In the subsequent test stages, different fabric reinforcements (uni- and bi-directional fabrics, woven fabrics, CSM layers) were considered for glass laminas only, and finally, a resultant laminate was designed and tested. Such an approach gives a great opportunity to create “tailor-made” laminates, as required in FRP bridge deck panels. Simultaneously with the laboratory tests, analytical calculations were performed using a few micromechanical models that aimed to determine engineering constants and strength parameters. Then, the results obtained from material testing and analytical calculations were compared, and conclusions on the compliance were drawn. Based on this validation, further analytical calculations may replace time-consuming laboratory tests and facilitate FRP deck design.

## 1. Introduction

Since the beginning of the 21st century, fiber-reinforced polymer (FRP) composites have become an integral part of the construction industry because of their high strength-to-weight ratio, resistance to corrosion and fatigue, enhanced durability, versatility, accelerated construction due to prefabrication, as well as lower maintenance and life-cycle costs [1,2,3]. Moreover, the properties of FRP structures can be tailored to meet the requirements by changing the fiber architecture. By varying the fiber type, the density within the matrix, the number of layers, and the orientation, the strength of an FRP structure can be customized in each direction. However, the design process is not in a code format, and individual FRP structural elements are designed on a case-by-case basis. A parametric study using the finite element method (FEM) is mostly conducted to examine design issues concerning the design of FRP structures [4,5,6,7,8,9,10]. Extensive research has also been conducted by the authors and collaborators on the modeling, optimization, and stiffness and strength evaluations of FRP bridge structures (i.e., decks or girders) in terms of both experimental investigations and FEM solutions [11,12,13,14,15,16]. In FE analysis the values of engineering constants of laminates are determined experimentally in the local directions of the material axes, and elaborated from a statistical point of view to obtain characteristic values. Although material testing is usually the most reliable approach to obtain credible data for design, it is costly, time-consuming, and thus impractical and very limited in its applicability for structural design purposes. For these reasons, the micromechanics approach, the technique used to obtain values of composite materials, is often adopted, whereby relatively accurate homogenization models are used to predict the equivalent properties of laminated composites [17,18,19,20].

The prediction of ply properties by micromechanics is well-defined, and the engineering constants of each layer can be computed from existing micromechanics models, such as rule of mixtures (LM), improved rule of mixtures (IL), and periodic microstructure (PM), whereby each layer is modeled as a homogeneous, linearly elastic, and generally orthotropic material [21,22,23]. Based on ply properties and lay-up, the apparent stiffnesses of the face laminate can be predicted by classical lamination theory (CLT) [24,25], and the micromechanical solution is also used for the evaluation of equivalent strength parameters of the laminates of FRP structures [18,26]. For the determination of strength parameters, micromechanics based on both analytical (linear model, improved fiber buckling method, fracture mechanics, strain amplification method, etc.) and empirical formulas were used. For simplified analysis and design optimization of FRP structures, it is useful to define representative strength parameters of the laminate in such an analytical way. To promote the wider acceptance of FRP structures, the development of standards and guidelines with reliable material parameters and computational methods that are easy to use for designers is needed.

In this paper, the material properties of the laminates to be used in a novel FRP bridge deck panel obtained by material testing have been compared to the respective properties predicted by micromechanics models. At the early stage of panel development, the application of all four of the most commonly used fibers was considered for panel laminates: aramid, basalt, carbon and glass. Four types of single ply (lamina) of AFRP, BFRP, CFRP and GFRP composites, respectively, made of biaxial fabrics and epoxy resin were tested in tension, shear and compression. After the most appropriate fibers had been chosen, the second stage of material testing was undertaken. This comprised the determination of the same strength parameters for GFRP (glass) plies only, but with different fabric reinforcement. Finally, in the third stage, the material properties of the GFRP laminates composed of different glass plies tested in the second stage were experimentally determined. Thus, the sequential design by testing was carried out to obtain “tailor-made” material properties, as required in FRP bridge deck panels [15,16,27,28]. Subsequently, micromechanical calculations were performed to determine analytically the same set of material properties to be compared and validated against the test results. The paper proposes a set of analytically determined and experimentally validated material properties of GFRP laminates, including tensile, shear and compressive strength, as well as elastic tensile and shear modules, to be used in panel design and further structural optimization. Conclusions drawn from the comparison of the experimentally and analytically determined material properties have been finally presented in the paper, comprising a validation of common analytical procedures used to facilitate reliable panel design. Furthermore, the results of comprehensive material testing can supplement the data available to date for FRP structural design, and may be collected for FRP design standardization worldwide [29].

## 2. Experiments

### 2.1. Materials

The four most popular fibers in the form of bidirectional fabrics were studied in the first stage of the research to compare the basic material properties of various fiber-reinforced epoxy composites. The following fabrics were used: aramid (B-A-470-1000), basalt (B-B-345-1000), carbon (B-C-600-1270) and glass (B-E-641-1300)—see Table 1.

When searching for the optimal stacking sequence of face laminates, the exact material properties of the single lamina are always required. Therefore, similar tests were carried out for the glass composites with different types of fiber reinforcement: unidirectional fabrics (U-E-600-1200), woven fabrics with reinforcement at the angle of ±45° (X-E-1210-1270), and woven fabrics with the same reinforcement system and an additional layer made of chopped strand mat (CSM) (X-S-E-1109-1270). Details of the fabrics used in this stage of the research are shown in Table 1.

The final stacking sequence of GFRP laminates was designed considering the test results for various glass composites, preliminary numerical simulations as well as the authors’ experience with the development of FRP deck panels [11,12,13,14,15,16]. The detailed structure of these face laminates is shown in Table 2 (face symmetry was assumed in the sandwich panel). Due to the limited availability of the target laminates, the X-E fabrics with a lower unit weight of 610 g/m^2^ (X-E-610-1270) were applied instead of 1200 g/m^2^ (X-E-1210-1270).

The epoxy system used in the research is Araldite LY1564 SP resin and Aradur 3489 hardener. The material properties of the hardened resin according to manufacturer information [30] are as follows: tensile strength—70 MPa; tensile modulus—3 GPa.

The 2 mm thick laminates were composed of reinforced plies, ranging from four to seven depending on the grammage, with epoxy resin fabricated using the hand lay-up method. The mold surface was cleaned and the releasing agent was applied. A thin layer of resin and hardener mixture was applied on the board. The fabrics were then filled with epoxy resin, and rolled to remove the entrapped air and to spread the mixture uniformly. Within 15 to 20 min, the epoxy resin was dried and set. A curing time of 3–4 h was given for the laminates to obtain good quality.

### 2.2. Methods

**Figure 1:**
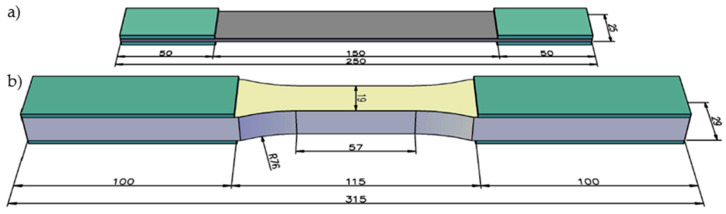
Geometry of typical tensile specimens (dimensions in mm); (**a**) rectangular specimen according to the ISO 527-4 standard; (**b**) dumbbell-shaped specimen according to the ASTM D638 standard.

**Figure 2:**
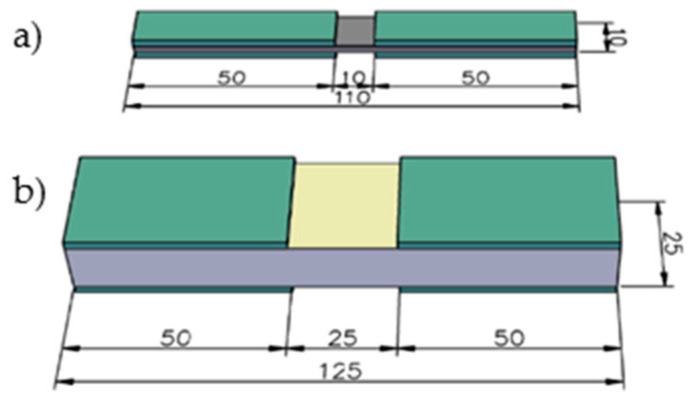
Geometry of typical compression specimens (dimensions in mm); (**a**) for the first and second stages of research, and (**b**) for face laminate.

In all stages of research, the tensile, shear and compression tests were carried out. The specimens in the first and second stages were tested according to standards ISO 527-1 [31] and ISO 527-4 [32]. The ISO 527-4 standard [32] allows specimens with thicknesses up to 10 mm to be tested in tension, which is less than in the case of the target laminate. Therefore, the dumbbell-shaped specimens were used following the ASTM D638 standard [33], which allows specimens with a thickness of up to 14 mm to be tested. The geometry of typical tensile specimens is presented in Figure 1. The shear tests were carried out according to the ISO 14129 [34] standard for in-plane shear by the ±45° tensile test, so the specimens for the shear test had the same geometry as in the tensile tests. The compression test was carried out according to the ISO 14126 standard [35], using the specimens with a length of 110 mm for the first and second stages of research, and with a length of 125 mm for face laminate—see Figure 2. All specimens had a constant cross-section.

The strengthening tabs were applied at the ends of the specimens to allow a smooth load transfer from the grip to the specimen, and to reduce the stress concentration and thus the shock wave stress effects. In all the tests, tabs of 2 mm thickness and 50 mm or 100 mm length were bonded on each side of the specimen.

For tensile tests, the Instron J1D hydraulic testing machine (Instron, Norwood, MA, USA) (1200 kN) equipment with Instron Bluehill software was used. The specimens were carefully positioned in the machine with their end faces exactly perpendicular to the longitudinal axis to get accurate results, as per the procedures given in the ISO 527-1 [31] and ISO 527-4 [32] standards for tensile tests and in the ISO 14129 [34] standard for in-plane shear by ±45° tensile tests. Longitudinal and transverse strains in each specimen were measured using a non-contact extensometer. The gauge length of 50 mm was used for the measurement of longitudinal strains, whereas 10 mm was used for the transverse ones. Similarly, for compressive tests, specimens were positioned and processed in a Matest testing machine (Matest, Arcore, Italy) (3000 kN) as per the procedure given in the ISO 14126 [35] standard for compression tests. This test was carried out using a dedicated fixture. The specimens were loaded with the displacement control at a speed of 2 mm/min for the tensile test and 1 mm/min for the compression test.

In each test, a series of ten specimens for each fabric type was tested. Unidirectional specimens were cut from laminates in the fiber direction. For laminates reinforced with the fabrics B-E-641-1300 and U-E-600-1200, and for face laminate, additional tensile and compression tests were carried out perpendicularly to the direction of the main reinforcement. The results obtained for the specimens sliding out from the machine jaws, damaged in the vicinity of the grip, or showing fabrication defects were excluded from the analysis.

### 2.3. Results and Discussion

The summarized test results of tensile, shear and compressive tests of composites with different fiber reinforcements are presented in Table 3.

**Figure 3:**
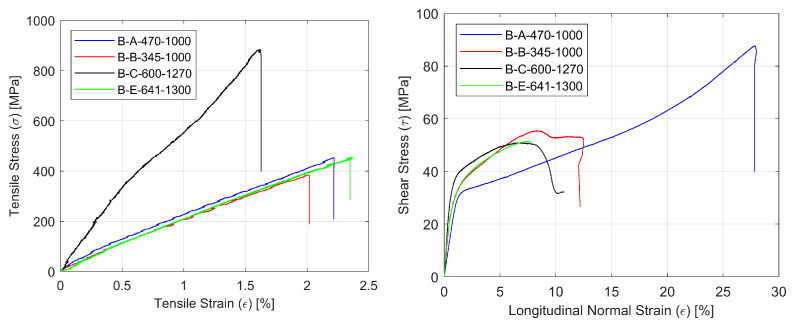
Representative curves for composite specimens with four types of reinforcement for tensile tests σ-ε (**left**) and the shear by tensile test τ-ε (**right**).

**Figure 4:**
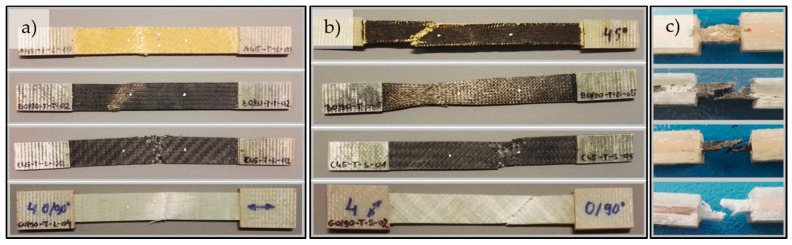
Typical failure modes of four types of reinforcement (from the top): B-A-470-1000 (aramid), B-B-345-1000 (basalt), B-C-600-1270 (carbon), and B-E-641-1300 (glass) for (**a**) tensile, (**b**) shear, and (**c**) compression.

Representative stress-strain curves for specimens with four types of reinforcement for the tensile (σ-ε) and shear by tensile test (τ-ε) are shown in Figure 3. Typical failure modes of the glass composite specimens under tension, shear and compression are presented in Figure 4.

Under tensile and shear by tension loading, in all specimens first matrix failure occurred, followed by fiber failure. Fibers fractured when their maximum strength was reached. These fibers’ fractures caused the ultimate failure in the form of the debonding of respective fibers and microcracking in the matrix. Fractures propagated spontaneously and the whole specimen raptured. Under compression load, the acceptable failure occurred in the gage section of the specimen, identifying them with the three-part identification HAT (“tHrough-thickness, At grip/tab, Top”), according to the standard [34].

As far as tensile behavior is concerned, the obtained values for laminates based on basalt, aramid and glass fibers are very similar. As expected, the carbon composite showed much higher strength and stiffness, but at the same time, its failure occurred at lower strain values. In contrast, the shear behavior of the aramid composite is quite different from that observed with the other three materials, which are very similar in terms of transverse shear strength (Figure 3). Finally, the compression behavior of the carbon composite showed the highest strength, but the compression strengths of the basalt and glass composites are two to three times higher than the aramid composite’s strength.

Summarizing the test results, glass composites were selected for further panel development. Aside from most favorable structural behavior, the choice was also determined by previous authors’ positive experiences with this material [11,12,13,14,15,16], technological considerations, the easy availability of glass products and the lowest price of the glass fabrics as compared to the remaining ones.

**Figure 5:**
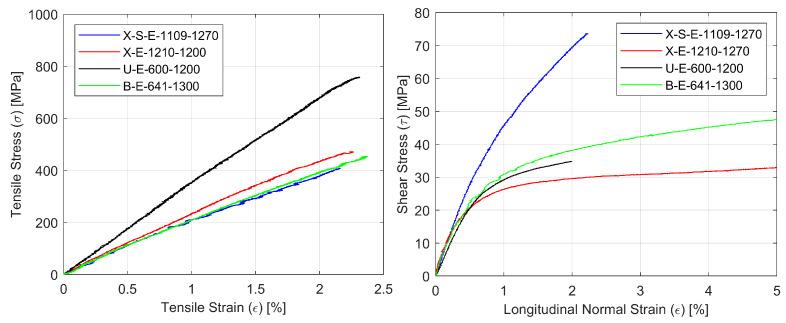
Representative curves for composite specimens with different glass fiber composites for tensile tests σ-ε (**left**) and shear by tensile test τ-ε (**right**).

**Figure 6:**
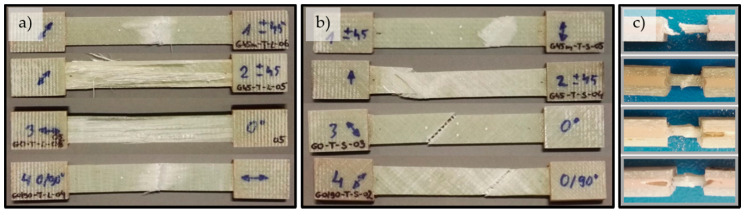
Typical tensile failure modes of glass composite specimens (from the top): X-S-E-1109-1270, X-E-1210-1270, U-E-600-1200, and B-E-641-1300 for (**a**) tensile, (**b**) shear, and (**c**) compression.

The summarized results of the tensile, shear and compressive tests of composites with different glass fiber reinforcements (the second stage of the research) are presented in Table 4. Representative stress-strain curves for specimens with all types of glass reinforcement for the tensile (σ-ε) and shear by tensile test (τ-ε) are shown in Figure 5. Typical failure modes of glass composite specimens under tension, shear and compression are presented in Figure 6.

As far as tensile behavior is concerned, the obtained values for laminates based on bidirectional fabrics B-E and woven fabrics X-E with reinforcement at the angle of ± 45° are very similar. As expected, the U-E fabric, with unidirectional reinforced composites, showed the highest strength and stiffness. The shear behavior of the X-S-E composites, with a ± 45° fiber direction and additional CSM layer, is quite different from that of the three remaining glass fabrics, which are very similar regarding shear strength. Finally, U-E unidirectional composites showed the highest strength in a compression test. The failure modes of glass fabric composites were similar to these described in the previous stage of the research.

The final stacking sequence of the target GFRP face laminates of the panel was designed considering the test results from this stage, i.e., the X-E-610-1270, U-E-600-1200 and B-E-641-1300 glass fabrics were chosen to be used in the final GFRP face laminates.

**Figure 7:**
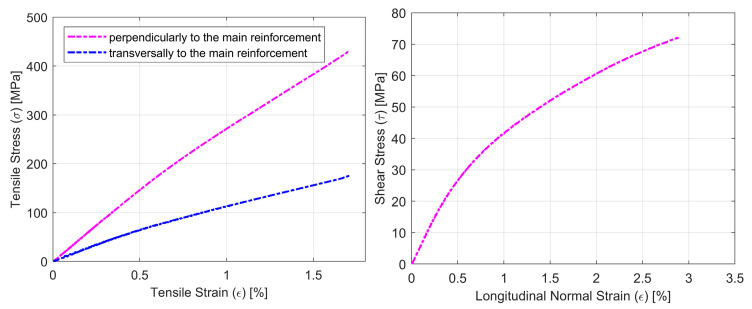
Representative curves for face laminate specimens at tension σ-ε (**left**) perpendicularly (1 direction) and transversally to main reinforcement (two directions) and shear by tensile test τ-ε (**right**).

**Figure 8:**
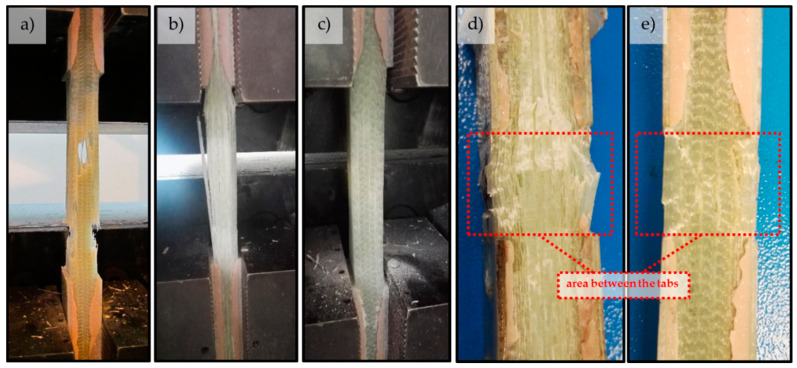
Examples of failure modes of GFRP face specimens in tension (**a**) perpendicularly and (**b**) transversally to the main reinforcement; (**c**) in shear (invisible to the naked eye) and compression (**d**) perpendicularly and (**e**) transversally to the main reinforcement.

The summarized test results of the target GFRP face laminate are presented in Table 5. Representative stress-strain curves for the tensile (σ-ε) and shear by tensile test (τ-ε) are shown in Figure 7. Typical failure modes of GFRP face laminate specimens under tension, shear and compression are presented in Figure 8.

The designed face laminate is a highly orthotropic material. The fibers are oriented mostly in direction 1 (approximately five of eight). Approximately two of eight of the other fibers are oriented in direction 2, and the remaining one in the other direction (mostly ± 45). This imbalance is observed in the results, where the average tensile strength and elastic modulus in the perpendicular direction (1) is over two times (2.3) higher than in the transverse direction (2): 434.08 MPa to 187.70 GPa and 30.67 MPa to 13.26 MPa, respectively. The ratio of compressive strength in directions 1 to 2 (f_1,c_/f_2,c_ = 1.93) is lower because the compressive strength is not only dependent on the pure fiber volume fraction, but also on the parameters of the resin. The addition of fibers oriented in the ± 45 direction significantly increased the material strength in the shear mode. As compared to the bidirectional 0/90 laminate, this was an increase of 58% for shear strength and 33% for shear modulus. Due to the mixed fiber orientation, the failure mode of specimens was more complex as compared with the failure mode of laminas in previous stages of research.

## 3. Micromechanics Calculation

### 3.1. Basic Assumptions

In analytical calculations, the FRP material is described using basic material engineering constants: elastic modulus (E), shear modulus (G) and Poisson’s ratio (ν). The second group comprises strength parameters: tensile (f_t_), compressive (f_c_) and shear strength (f_v_). In the case of heterogeneous laminates consisting of a series of laminas with different fiber orientations, the experimental determination of these parameters is a costly and time-consuming process. Alternatively, it is possible to conduct analytical calculations based on the properties of two basic laminate components: fibers and resin. In Table 6 the engineering constants and strength parameters for all types of fibers and epoxy resins are presented as given in the internal draft version of the new Eurocode on the design of fiber-reinforced polymer structures [29]. The values included in Table 6 are based on the material properties reported in the literature, and are not necessarily concomitant. Additionally, for the fibers, the reduction in tensile strength due to microdamage during the production of fibers was taken into account, following Ref. [36] (50% reduction for glass fibers, 31% for aramid and 20% for carbon) and Ref. [37] (41% reduction for basalt fibers). The reduced strength was named as the effective tensile strength, f_f,t_. The pure compressive strength of fibers is not decisive, as it is very unlikely that the failure of entire laminates is caused by the failure of compressed fibers alone. The loss of stability or shear failure in or out of the plane of the laminate occurs much more often. For this reason, the draft in Ref. [29] does not provide these values. The same explanation applies to the shear strength of the fibers, as the shear strength of the laminate is generally determined by the shear of the matrix, not the fibers.

It is worth noting that the constants for some fibers show dependence, as for an isotropic material (glass fibers) or a fairly isotropic one (basalt fibers). There is no such correlation for the aramid and carbon fibers.

The measured thicknesses of individual specimens for all laminates allowed for determining the fiber volume fraction (V_f_) based on the Formula (1):

(1) $$V_{f}=\frac{\sum \omega_{f}}{\rho_{f}\;t}$$

where

ω_f_—the weight per unit area of all the fabric in the laminate;
ρ_f_—density of the fiber material;
  t—thickness of the laminate.

The fiber volume fraction (V_f_) values obtained for all specimens are presented in Table 7.

**Figure 9:**
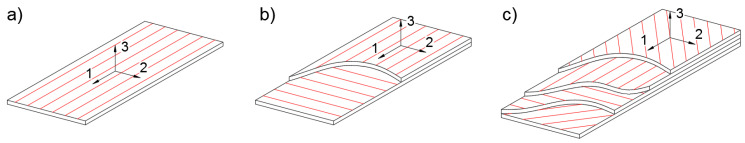
Designations and orientations of the coordinate system for layers: unidirectional reinforced (**a**), bidirectional reinforced (**b**) and face laminate (**c**).

For the sake of unification, one coordinate system was adopted, regardless of the number of layers, their orientation and the types of fabrics and materials (Figure 9).

### 3.2. Engineering Constants

The models that describe the laminate concerning its components are called micromechanical models, and the calculations of material properties conducted on their basis are called micromechanical analyses of composites. The micromechanical material models of the laminate may be empirical, analytical, or numerical. For the determination of engineering constants (E_1_, E_2_, ν_12_, G_12_), three models were applied: the linear model (otherwise known as rule of mixtures and inverse rule of mixtures), a combined model called the improved linear model (otherwise known as improved rule of mixtures) and the periodic microstructure model. The formulas used to determine the engineering constants according to three previously mentioned micromechanical models are given in Appendix A and the results are summarized in Table 8.

### 3.3. Strength Parameters

The determination of the strength of an FRP composite laminate can vary significantly depending on the direction of the load, concerning the direction of the fibers and the type of force: compressive or tensile. The five most common load cases, related formulas and micromechanical models are described in Appendix B. The strength parameters determined on their basis for individual specimens are summarized in Table 9. The assumed values of the parameters and coefficients used in the calculations (Formulas (A16)–(A26)) are also listed in Table 9.

## 4. Comparison between Experiment and Micromechanics

**Figure 10:**
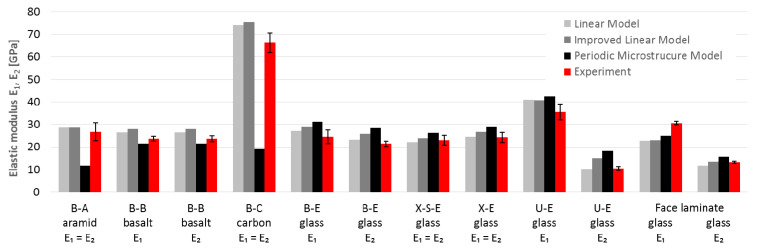
Comparison of elastic modulus E_1_ and E_2_ obtained by calculation and experiment.

**Figure 11:**
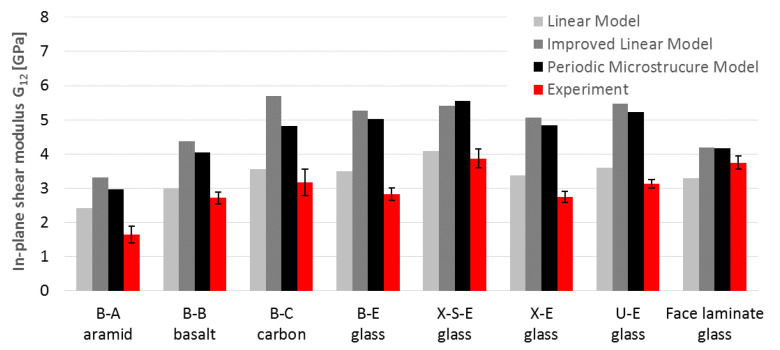
Comparison of in-plane shear modulus G_12_ obtained by calculation and experiment.

**Figure 12:**
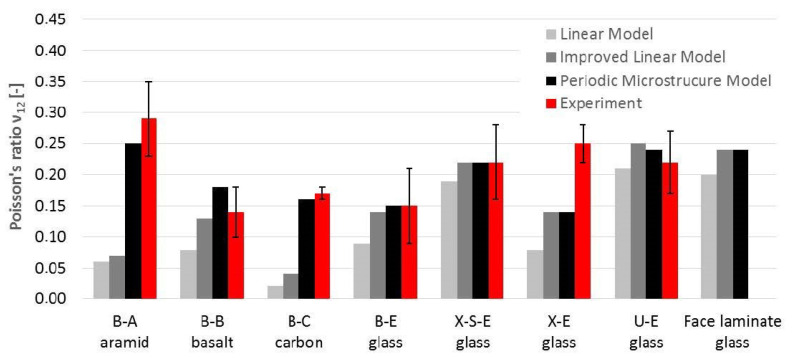
Comparison of Poisson’s ratio ν_12_ obtained by calculation and experiment.

The comparison of the engineering constants obtained from the experiment (Table 3, Table 4 and Table 5) with the results of micromechanical calculations (Table 8) is shown in Figure 10 (elastic moduli), Figure 11 (in-plane shear modulus) and Figure 12 (Poisson’s ratio).

Based on these charts, it may be concluded that:

- The E_1_ and E_2_ values for glass laminates (U-E, B-E, X-E and X-S-E) based on two analytical micromechanical models are in very good compliance with average test results (97–115% for ML, 104–117% for IL). The only exception was E_2_ for face laminate, which is 144% of the IL-calculated value. Besides this, 50% of the calculated values lay in the standard deviation range of test results;
- The periodic microstructure approach gave higher values of 114–132% for all specimens except E_2_ for U-E (175%). The calculation of face laminates showed wider scatter for both moduli E_1_ (75% for ML and IL, 81% for PM) and E_2_ (89%, 103% and 118% respectively). All calculated results lay outside of the range of the results’ standard deviation;
- Comparisons of E_1_ and E_2_ values for other types of fibers (B-A, B-B and B-C) were quite similar, with compliance in the range of 107–119% for analytical models LM and IL. Only one of the three results was in the range of the results’ standard deviation. The PM model was inappropriate for anisotropic fibers (B-A and B-C) because of its 44% and 29% compliance with test results for aramid and carbon fibers, respectively. For basalt fibers the compliance was satisfactory, i.e., 91%;
- G_12_ values for all laminates based on LM were only a little overestimated (115% on average). Two further models (IL and PM) overestimated G_12_ for all laminates much more, on average 167% for IML (111–201%) and 157% for PM (111–180%). Only 8% of the results lay in the standard deviation range of the test results (88% of the calculated results were overestimated);
- The shear modulus values obtained in the tests were lower than those calculated on both micromechanical models: IL and PM. This could be the result of the adopted test method for the shear test. It is widely believed that the values obtained from tests following the procedures of the PN-EN ISO 14129 standard [34] are minimal. Other types of shear testing are not as popular and standardized because they require specialized overlays and equipment, as well as specific specimen forms. However, recent documents [38] and as-yet unpublished drafts of new Eurocode [29] indicate other methods as more realistic;
- The calculated values of Poisson’s ratio ν_12_ were the most underestimated values among the engineering constants. Only PM values gave satisfactory result with an average of 96% compliance (in the range of 56–129%). The LM showed lower consistency with 52% compliance on average (in the range of 12–95%) and IL with 72% compliance on average (in the range of 24–114%). About 48% of the calculated values were in the standard deviation ranges of the test results, and 48% of the calculated results were underestimated.

**Figure 13:**
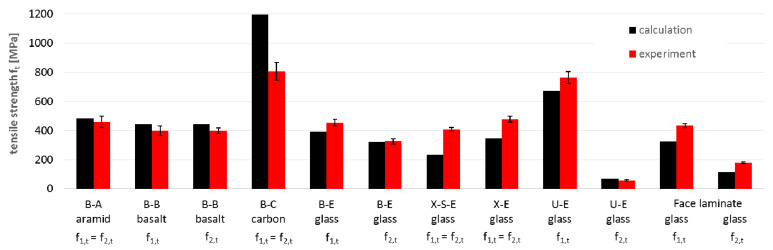
Comparison of tensile strength f_1,t_ and f_2,t_ obtained by calculation and experiment.

**Figure 14:**
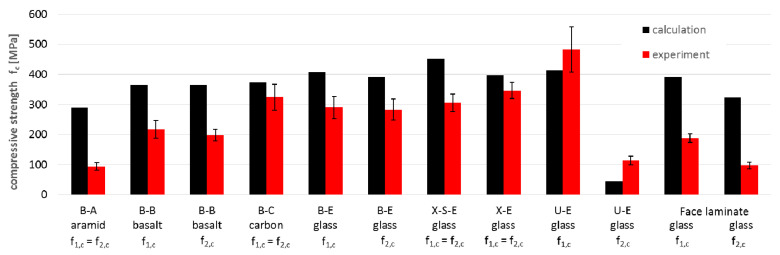
Comparison of compression strength f_1,c_ and f_2,c_ obtained by calculation and experiment.

**Figure 15:**
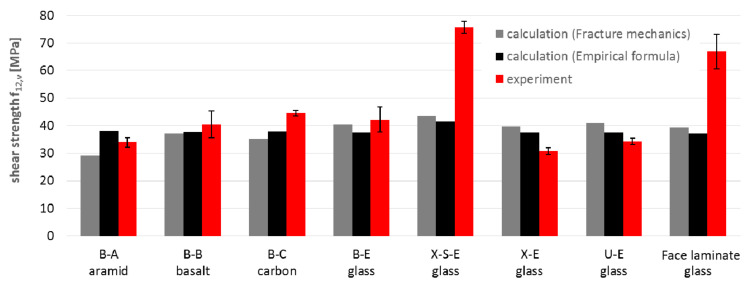
Comparison of shear strength f_12,v_ obtained by calculation and experiment.

Similarly, the values of the strength parameters were compared between experimental (Table 3, Table 4 and Table 5) and calculated ones (Table 9). The results of the comparisons are presented for tensile strength (Figure 13), compressive strength (Figure 14) and shear strength (Figure 15).

Based on these charts, it may be concluded that:

- The average compliance for all 12 tensile strength values f_t_ is 95%, but single values varied from 57% to 148%. The tensile strength for carbon fibers is the most overestimated (148%), and it may prove that the actual strength of carbon fibers is lower than assumed (Table 7) and that reduction is higher than the adopted 20%. In turn, the most underestimated of the calculations is the tensile strength of the X-S-E laminate, which may indicate a higher strength of the CSM layer than that which resulted from the adopted micromechanical model for the CSM layer. The tensile strength for the rest of the specimens did not differ significantly from the experimental results (about ±10%);
- The compressive strength showed noticeably lower compliance. The average compliance for the 12 test values was 165% (Figure 14). The greatest overestimation by calculations occurred for aramid and basalt fibers (about 300% and 175%, respectively). The reasons can come both from the adopted material constants as well as from the adopted micromechanical models for these types of fibers. A similarly high overestimation of the compressive strength occurred for the face laminate. This is related to the fact that in-plane shear also contributes to the failure of the multilayer, thick, compressed laminate, which it was not possible to capture in the adopted micromechanical models. After omitting these two groups, the results for the remaining glass and carbon laminates showed a low average discrepancy of approximately 10%. For the U-E laminate the calculated compressive strength in both directions was lower than the results of the experiment;
- The shear strength values showed high compliance between calculation and experiment, with the average value for all specimens being as much as 90% for both micromechanical models. Significant discrepancies can be observed in the case of the X-S-E laminates with CSM layers, whose share in the shear strength was underestimated in both micromodels. A similar underestimation applies to the face laminate, and is related to the mismatch of micromechanical models (which are formulated generally to the individual, often unidirectionally reinforced lamina) to the analysis of multilayer laminates. Besides this, micromechanical models are based on the assumption that the material works linearly, while specimens subjected to shear showed a strong non-linearity. For X-S-E and face laminates, the τ-ε plots do not show initial linearity with a fracture point, as in the case of other types of fibers (Figure 5 and Figure 7). This proves the different nature of the behavior of this type of laminate, and therefore the need for a different calculation approach than with the others fibers is revealed.

## 5. Conclusions

Comprehensive material tests comprising almost 250 individual specimens were conducted to determine the basic material properties of individual laminas and laminates reinforced with four types of fibers (carbon, glass, basalt, and aramid) embedded in an epoxy matrix. The obtained test results were used to find the most suitable materials for sandwich panel face laminates, as well as to validate the results of analytical calculations performed in parallel using a few micromechanical models. This research, based on the comparison of various laminates characterized by experimental and calculated material properties, may be concluded as follows.

The comparison of laminates bi-directionally reinforced with different (aramid, basalt, carbon and glass) fibers did not show any major differences in engineering constant. The exception was carbon fiber-reinforced laminates, which were approximately 2.5 times stiffer (E_1_) as compared to the respective values of the remaining composites. Aramid fiber composites showed a slightly lower shear modulus. Taking into account the obtained comparison as well as technological and economic factors, it was decided to conduct further research on glass fiber composites only, as the most promising material for the mass production of load-bearing deck panels.

The second phase of the research involved material tests carried out on laminas made of various types of glass reinforcement: uni- and bi-directional fabrics as well as a mat. The research showed that there are no major differences between the strength parameters of the glass laminas depending on the reinforcement type, thus it was decided not to use XSE fabrics in target face laminates. In these, the adopted stacking sequence of fabrics allowed us to design the optimal orthotropic material with material parameters between the lamina with bi-directional and uni-directional fibers. The use of 45 degree fabric spacers made it possible to achieve high shear strength, exceeding the shear strength of the bi-directional fabrics alone. This was only slightly lower (12%) than the shear strength for XSE laminas, despite the absence of CSM mats in the target face laminate.

A total of seven micromechanical models was applied to calculate the material properties of GFRP laminates and to compare them with the experimentally determined counterparts. The inevitable discrepancies between experimental and calculated material properties did not exceed approximately 20% in most cases. The improved linear model is the most accurate model to calculate the engineering constants of glass fibers with an average compliance of 91%.

From a practical point of view, the most important conclusion of this research comes from the comparison of the test results with the calculations carried out according to an unpublished draft of the standard [29]. As the comparison showed, the use of the proposed material parameters for the FRP laminate components allows for obtaining satisfactory compatibility for all types of fibers and types of reinforcement. Having a database of reliable material test results, it is possible to skip some of the future tests when an appropriate structure of laminates in load-bearing elements is sought.

## Figures and Tables

**Table 1:** Details of fabrics used in the first and second stages of the research.

Stage	Fabric Type	Material	Fiber Direction	Unit Weight	Lamina Thickness
				(g/m^2^)	(mm)
1	B-A-470-1000 (aramid)	Aramid 316 tex	0/90	470 ± 5%	2.46 ± 0.10
	B-B-345-1000 (basalt)	Basalt 16/9 F/cm	0/90	345 ± 25	2.01 ± 0.03
	B-C-600-1270 (carbon)	Carbon 800 tex	0/90	600 ± 5%	1.64 ± 0.06
	B-E-641-1300 (glass)	E-Glass 1200 tex E-Glass 600 tex	0/90	641 ± 5%	2.55 ± 0.04
2	X-S-E-1109-1270 (glass)	E-Glass 600 tex E-Glass 68 tex	±45	1109 ± 5%	2.53 ± 0.04
	X-E-1210-1270 (glass)	E-Glass 1200 tex	±45	1210 ± 5%	2.52 ± 0.03
	U-E-600-1200 (glass)	E-Glass 1200 tex E-Glass 68 tex	0	600 ± 5%	2.32 ± 0.02

**Table 2:** Structure of target face laminate.

Layer	Fabric Type	Fiber Direction	Fabric Thickness	Unit Weight	No. Fabrics	Layer Thickness	Unit Weight
			(mm)	(g/m^2^)		(mm)	(g/m^2^)
1	B-E-641-1300	0/90	0.45	641	6	2.70	3846
2	X-E-610-1270	±45	0.43	610	1	0.43	610
3	U-E-600-1200	0	0.42	600	5	2.10	3000
4	X-E-610-1270	±45	0.43	610	1	0.43	610
5	U-E-600-1200	0	0.42	600	5	2.10	3000
6	X-E-610-1270	±45	0.43	610	1	0.43	610
7	U-E-600-1200	0	0.42	600	5	2.10	3000
8	X-E-610-1270	±45	0.43	610	1	0.43	610
9	B-E-641-1300	0/90	0.45	641	6	2.70	3846
In total						13.42	19,132

**Table 3:** Test results in the first stage of the research.

Laminate	Tensile				Shear		Compressive
	Strength f_1,t_	Ult. Stain ε_1,u_	Elastic Modulus E_1,t_	Poisson’s Ratio ν_12_	Strength f_12,v_	Modulus G_12_	Strength f_1,c_
	(MPa)	(%)	(GPa)	(-)	(MPa)	(GPa)	(MPa)
B-A-470-1000 (aramid)	458.80 ± 37.85	2.33 ± 0.17	26.80 ± 4.07	0.29 ± 0.06	33.93 ± 1.78	1.65 ± 0.24	93.79 ± 13.10
B-B-345-1000 (basalt)	399.13 ± 31.61	2.10 ± 0.21	23.72 ± 1.09	0.14 ± 0.04	40.49 ± 4.95	2.72 ± 0.17	217.24 ± 29.32
B-C-600-1270 (carbon)	806.68 ± 62.01	1.65 ± 0.14	66.31 ± 4.31	0.17 ± 0.00	44.53 ± 1.09	3.17 ± 0.39	323.90 ± 43.39
B-E-641-1300 (glass)	454.39 ± 23.11	2.29 ± 0.11	24.65 ± 3.03	0.15 ± 0.03	42.19 ± 4.63	2.83 ± 0.18	290.09 ± 36.88

**Table 4:** Test results in the second stage of the research.

Laminate/ Direction		Tensile				Shear		Compressive
		Strength f_i,t_	Ult. Stain ε_i,u_	Modulus E_i,t_	Poisson’s Ratio ν_12_	Strength f_12,v_	Modulus G_12_	Strength f_i,c_
		(MPa)	(%)	(GPa)	(-)	(MPa)	(GPa)	(MPa)
X-S-E-1109-1270	1, 2	408.55 ± 14.15	2.22 ± 0.08	22.97 ± 2.15	0.22 ± 0.06	75.62 ± 2.12	3.87 ± 0.27	305.48 ± 28.75
X-E-1210-1270	1, 2	477.39 ± 19.77	2.28 ± 0.12	24.25 ± 2.27	0.25 ± 0.03	30.82 ± 1.23	2.75 ± 0.16	346.48 ± 26.64
U-E-600-1200	1	765.92 ± 40.39	2.30 ± 0.13	35.48 ± 3.45	0.24 ± 0.02	34.24 ± 1.12	3.14 ± 0.12	482.89 ± 75.55
	2	58.29 ± 4.77	1.15 ± 0.23	10.46 ± 0.90	0.22 ± 0.05			114.16 ± 14.72
B-E-641-1300	1	454.39 ± 23.11	2.29 ± 0.11	24.65 ± 3.03	0.15 ± 0.03	42.19 ± 4.63	2.83 ± 0.18	290.09 ± 36.88
	2	325.95 ± 19.71	1.98 ± 0.10	21.44 ± 1.26	0.15 ± 0.06			283.02 ± 34.86

**Table 5:** Test results for target GFRP face laminate.

Laminate/ Direction		Tensile			Shear		Compressive
		Strength f_i,t_	Ult. Stain ε_i,u_	Modulus E_i,t_	Strength f_12,v_	Modulus G_12_	Strength f_i,c_
		(MPa)	(%)	(GPa)	(MPa)	(GPa)	(MPa)
**GFRP laminate**	1	434.08 ± 14.20	1.71 ± 0.13	30.67 ± 0.71	66.80 ± 6.32	3.75 ± 0.19	187.70 ± 14.50
	2	177.47 ± 5.08	1.83 ± 0.15	13.26 ± 0.44			96.91 ± 11.01

**Table 6:** The material constants and strength parameters for all types of fibers and epoxy resin [29].

Component	Material Constants				Strength Parameters		
	Elastic Modulus		In-Plane Shear Modulus	Poisson’s Ratio	Nominal Tensile Strength	Effective Tensile Strength	Compressive Strength
	E_1_	E_2_	G_12_	ν_12_	f_t,brutto_	f_t_	f_c_
	(GPa)	(GPa)	(GPa)	(-)	(MPa)	(MPa)	(MPa)
Glass fibers	74	74	30	0.25	2500	1250	-
Aramid fibers	130	10	12	0.35	3600	2484	-
Basalt fibers	90	90	22	0.31	3000	1773	-
Carbon fibers	230	20	16	0.20	4900	3920	-
Epoxy resin	3.0	3.0	1.6	0.40	35	35	80

**Table 7:** Fiber volume fraction (V_f_).

	Series Identifier (Fiber’s Material)							
	B-A-470-1000	B-B-345-1000	B-C-600-1270	B-E-641-1300	X-S-E-1109-1270	X-E-1210-1270	U-E-600-1200	Face Laminate
	Aramid	Basalt	Carbon	Glass	Glass	Glass	Glass	Glass
V_f_	39	50	61	58	56 ^1^/43 ^2^	56	60	41

^1^—laminate with continuous fibers, ^2^—laminate with chopped fibers (chopped strand mat, CSM).

**Table 8:** The calculated material constants for all types of fibers.

Laminate	Longitudinal Elastic Modulus			Transverse Elastic Modulus			In-Plane Shear Modulus			Poisson’s Ratio		
	E_1_ (GPa)			E_2_ (GPa)			G_12_ (GPa)			ν_12_ (-)		
	LM ^1^	IL ^2^	PM ^3^	LM ^1^	IL ^2^	PM ^3^	LM ^1^	IL ^2^	PM ^3^	LM ^1^	IL ^2^	PM ^3^
B-A-470-1000 (aramid)	28.57	28.67	11.91	28.57	28.67	11.91	2.42	3.31	2.97	0.06	0.07	0.25
B-B-345-1000 (basalt)	26.42	28.10	21.59	26.42	28.10	21.59	2.98	4.37	4.05	0.08	0.13	0.18
B-C-600-1270 (carbon)	74.08	75.40	19.17	74.08	75.40	19.17	3.55	5.69	4.82	0.02	0.04	0.16
B-E-641-1300 (glass)	27.07	28.95	31.29	23.36	25.83	28.34	3.49	5.27	5.03	0.09	0.14	0.15
X-S-E-1109-1270 (glass)	22.20	23.87	26.24	22.20	23.87	26.24	4.08	5.42	5.56	0.19	0.22	0.22
X-E-1210-1270 (glass)	24.65	26.68	29.08	24.65	26.68	29.08	3.38	5.07	4.84	0.08	0.14	0.14
U-E-600-1200 (glass)	40.94	40.76	42.44	10.37	15.11	18.31	3.59	5.48	5.23	0.21	0.25	0.24
Face laminate (glass)	22.87	23.06	24.96	11.74	13.60	15.64	3.30	4.18	4.17	0.20	0.24	0.24

^1^—Linear Model, ^2^—Improved Linear Model, ^3^—Periodic Microstructure Model.

**Table 9:** Strength parameters for all specimens calculated according to different micromechanical theories.

Laminate	Longitudinal Direction		Transverse Direction					In-Plane	
	Tensile	Comp.	Tensile		Compressive			Shear	
	f_1,t_ (MPa)	f_1,c_ (MPa)	f_2,t_ (MPa)		f_2,c_ (MPa)			f_12,v_ (MPa)	
	LM ^1^	IFB ^2^	LM ^1^	FM ^3^	IFB ^2^	SA ^4^	EF ^5^	FM ^6^	EF ^7^
B-A-470-1000 (aramid)	484	289	484	-	289	-	-	29.1	38.1
B-B-345-1000 (basalt)	443	366	443	-	366	-	-	37.3	37.6
B-C-600-1270 (carbon)	1196	374	1196	-	374	-	-	35.3	37.9
B-E-641-1300 (glass)	391	407	320	-	407	-	-	40.4	37.5
X-S-E-1109-1270 (glass)	234	451	234	-	451	-	-	43.6	41.5
X-E-1210-1270 (glass)	348	398	348	-	398	-	-	39.7	37.5
U-E-600-1200 (glass)	671	414	69.0	56.2	-	44.6	49.8	40.9	37.5
Face laminate (glass)	323	392	113	-	392	-	-	39.3	37.2

^1^—Linear model, ^2^—Improved fiber buckling method, α_σ_ assumed to be 3 deg, ^3^—Fracture mechanics, adopted values according to Ref. [36]: G_Ic_ = 360 J/m^2^; t_t_ = 0.6 (glass and basalt) or 0.8 mm (carbon and aramid); material constants from Table 8 (LM), ^4^—Strain amplification method, ^5^—Empirical formulas, adopted values: V_u_ = 0.02, σ_r,c_ = 80 MPa from Table 6, ^6^—Fracture mechanics, adopted values according to Ref. [36]: G_2c_ = 220 J/m^2^; t_t_ = 0.6 (glass and basalt) or 0.8 mm (carbon and aramid); G_12_ from Table 8 (LM), ^7^—Empirical formulas, V_u_ assumed to be 2%; τ_r_ was calculated on test results and was adopted as 60.1 MPa.

## Data Availability

Data sharing is not applicable to this article.

## Appendix A. Engineering Constants

The simplest micromechanical models of the single lamina are the Voigt linear model (otherwise known as the rule of mixtures) [39] and the Reuss model (commonly referred to as the inverse rule of mixture) [40]. Together, both models (abbreviated as LM) allow us to determine a set of engineering constants for unidirectionally reinforced lamina based on Formulas (A1)–(A5) (index f stands for fiber and r stands for resin):

(A1) $$E_{1}=E_{f,1}{\;V}_{f}+E_{r}\;\left(1-V_{f}\right)$$

(A2) $$E_{2}=\frac{E_{f,2}{\;E}_{r}}{E_{r}{\;V}_{f}+E_{f,2}\;\left(1-V_{f}\right)}$$

(A3) $$G_{12}=\frac{G_{f}\;G_{r}}{G_{r}{\;V}_{f}+G_{f}\;\left(1-V_{f}\right)}$$

(A4) $$\nu_{12}=\nu_{f}{\;V}_{f}+\nu_{r}\;\left(1-V_{f}\right)$$

These simple analytical micromechanical models have many newer variations, both analytical and empirical, which are developed to get a more accurate match of the calculation results with the results of the experimental tests. The most popular, the so-called improved rule of mixture [41] (abbreviated as IL) was used as an alternative to LM. This theory modifies the formulas for the transverse direction to fibers, which is often underestimated by the LM. The formulas for engineering constants in the transverse direction are given in Formulas (A5)–(A8):

(A5) $$\eta_{2}=\frac{0.2}{1-\nu_{r}}\;\left(1.1-\sqrt{\frac{E_{r}}{E_{f}}}+\frac{3.5{\;E}_{r}}{E_{f}}\right)\;\left(1+0.22{\;V}_{f}\right)$$

(A6) $$E_{2}=\frac{E_{f,2}{\;E}_{r}\;\left(V_{f}+\eta_{2}\;\left(1-V_{f}\right)\right)}{E_{r}{\;V}_{f}+E_{f,2}{\;\eta }_{2}\;\left(1-V_{f}\right)}$$

(A7) $$\eta_{12}=0.28+\sqrt{\frac{E_{r}}{E_{f}}}$$

(A8) $$G_{12}=\frac{G_{f}{\;G}_{r}\;\left(V_{f}+\eta_{12}\;\left(1-V_{f}\right)\right)}{G_{r}{\;V}_{f}+G_{f}{\;\eta }_{12}\;\left(1-V_{f}\right)}$$

Finally, the empirical periodic microstructure model was used (abbreviated as PM), which takes into account the manner in which the fibers are distributed in the composite, instead of just their volume ratio [1]. It is assumed that the fibers in the composite are arranged in a structured, periodical manner. Based on this assumption, it is possible to use the Fourier series to determine all elements of the laminate stiffness tensor. According to Ref. [1], the engineering constants of the lamina may be determined directly from the following Formulas (A9)–(A12):

(A9) $$E_{1}=C_{11}-\frac{2{\;C}_{12}{}^{2}\;}{C_{22}+C_{33}}$$

(A10) $$E_{2}=\frac{\left(2{\;C}_{11}C_{22}+2{\;C}_{11}C_{23}-4{\;C}_{12}{}^{2}\right)\;\left(C_{22}-C_{23}+2{\;C}_{44}\right)\;}{3{\;C}_{11}{\;C}_{22}+C_{11}C_{23}+2{\;C}_{11}C_{44}-4{\;C}_{12}{}^{2}}$$

(A11) $$G_{12}=C_{66}$$

(A12) $$\nu_{12}=\frac{{\;C}_{12}\;}{C_{22}+C_{23}}$$

where C_ij_ are elements of the stiffness tensor for lamina.

Lamina reinforced with CSM can be treated as a quasi-isotropic material in its plane. For determining the engineering constants, the CSM lamina is idealized as a laminate with a number of thin, unidirectional layers, each with a different fiber orientation in the range 0–180°. Then, engineering constants for the whole CSM-reinforced lamina are averaged. Finally, the engineering constants of the CSM layer can be derived according to Ref. [36] from Formulas (A13)–(A15):

(A13) $$E_{\mathrm{csm}}=\frac{3{\;E}_{1}}{8}+\frac{5{\;E}_{2}}{8}$$

(A14) $$G_{\mathrm{csm}}=\frac{1{\;E}_{1}}{8}+\frac{1{\;E}_{2}}{4}$$

(A15) $$\nu_{\mathrm{csm}}=\frac{E_{\mathrm{csm}}}{2{\;G}_{\mathrm{csm}}}-1$$

where

E_1_, E_2_—the elastic modulus of the virtually unidirectional layer with the same V_f_ as the real CSM layer, determined from, for example, the Formulas (A1) and (A2) or another micromechanical model.

## Appendix B. Strength Parameters

### Appendix B.1. Longitudinal Tensile Strength

The simplest model for the determination of tensile strength for a continuous fiber-reinforced composite is derived by assuming that all the fibers have the same tensile strength, behave linearly and are brittle with respect to the matrix. Based on that assumption, the tensile strength of unidirectionally reinforced lamina f_t_ is calculated from Formula (A16):

(A16) $$f_{1,t}={\;f}_{f,t}\;\left(V_{f}+\frac{E_{r}}{E_{f}}\left(1-V_{f}\right)\right)$$

where

f_f,t_—tensile strength of fibers.

Observing that the ratio of the elastic modulus (E_r_ to E_f_) ranges from 1 to 4% (Table 7, depending on the type of fibers), the above equation can be simplified. Additionally, Formula (A16) can be expanded to multilayer laminates, if only fibers oriented in the direction of the tensile forces are considered. After these operations, the formula for strength in the i-direction will take the form (A17):

(A17) $$f_{i,t}=\;\frac{\rho_{f,i}{\;V}_{f}{\;f}_{f,t}}{\rho_{f}}$$

where

ρ_f,i_—the weight of fibers in the i-direction,
ρ_f_—total weight of fibers.

### Appendix B.2. Longitudinal Compressive Strength

The compressive strength can be obtained from Formulas (A16) and (A17) using the compressive strength of the fibers f_f,c_ instead of the tensile strength f_f,t_. However, this is not a real value, as most compressed laminates are failed by shearing or a fiber buckling. In addition, the perfect parallel alignment of the fibers is not possible in reality. The analytical solution, so-called the improved fiber buckling method [42], determines compressive strength f_1,c_ by the Formula (A18):

(A18) $$f_{1,c}={\;G}_{12}{\left(1+\;\frac{4.76{\;G}_{12}{\;\alpha }_{\sigma }}{f_{12,v}}\right)}^{-0.69}$$

where

G_12_—shear modulus of laminate,
α_σ_—standard deviation of fiber misalignment,
f_12_—shear strength of the laminate.

### Appendix B.3. Transverse Tensile Strength

The failure of lamina without fibers in the direction of tension (transversal direction of the unidirectional lamina) occurs when a crack appears and propagates, so it can be described on the basis of fracture mechanics [36]. The tensile strength transverse to the fiber direction f_2,t_ can be estimated from the Formulas (A19) and (A20):

(A19) $$f_{2,t}=\;\sqrt{\frac{G_{\mathrm{Ic}}}{1.12^{2}\;\pi \;\frac{t_{t}}{4}{\;\Lambda }_{22}}}$$

(A20) $$\Lambda_{22}=2\;\left(\frac{1}{E_{2}}-\frac{\nu_{12}{}^{2}{\;E}_{2}{}^{2}}{E_{1}{}^{3}}\right)$$

where

G_Ic_—fracture toughness in crack opening mode I based on fracture mechanics,
t_t_—transition thickness.

### Appendix B.4. Transverse Compressive Strength

The transverse compressive strength f_2,c_ could be obtained from an analytical method based on the strain-magnification factor [43] and calculated from Formula (A21):

(A21) $$f_{2,c}=E_{2}\;\varepsilon_{r,c}\;\left[1-\sqrt{\frac{4{\;V}_{f}}{\pi }}\left(1-\frac{E_{r}}{E_{f,2}}\right)\right]$$

where

ε_rc_—maximum shortening of a resin in compression,
E_f,2_—fiber’s elastic modulus in the transversal direction.

The alternative way to determined transverse compressive strength f_2,c_ is empirical Formula (A22) [36,44]:

(A22) $$f_{2,c}=\sigma_{r,c}\;\left[1-\sqrt{\frac{4{\;V}_{v}}{\pi \;\left(1-V_{f}\right)}}\right]\;\left[1+\left(V_{f}-\sqrt{V_{f}}\right)\left(1-\frac{E_{r}}{E_{f,2}}\right)\right]$$

where

σ_r,c_—compressive strength of the resin,
V_v_—void volume fraction.

### Appendix B.5. In-Plane Shear Strength

Shear failure in the plane of the lamina occurs when a crack between the fibers and the matrix propagates along the fiber direction. Therefore, shear strength is a problem of fracture mechanics [36]. The following formula can be used to predict the shear strength in plane f_12_ of the unidirectional lamina (A23):

(A23) $$f_{12,v}=\;\sqrt{\frac{G_{\mathrm{IIc}}}{1.12^{2}\;\pi \;\frac{t_{t}}{4}{\;\Lambda }_{44}}}$$

where

G_IIc_—fracture toughness in crack opening mode II based on fracture mechanics.

An empirical Formula (A24), similar to (A22), can be used as an alternative [36,44]:

(A24) $$f_{12,v}=\tau_{r}\;\left[1-\sqrt{\frac{4{\;V}_{v}}{\pi \;\left(1-V_{f}\right)}}\right]\;\left[1+\left(V_{f}-\sqrt{V_{f}}\right)\left(1-\frac{G_{r}}{G_{f}}\right)\right]$$

where

τ_r_—shear strength of resin.

### Appendix B.6. CSM Layer

The strength parameters for the CSM layer are determined assuming that the CSM lamina is isotropic in its plane. With this assumption, the tensile strength f_t,csm_ can be determined from the Formulas (A25) and (A26) [36,45]:

(A25) $$f_{t,\mathrm{csm}}=\{\begin{array}{cc} \frac{4\;{f^{\prime }}_{12,v}}{\pi }\left(1+\ln\left(\alpha \right)\right) & \mathrm{for}\;\alpha \;>\;1\\ \frac{4\;{f^{\prime }}_{12,v}}{\pi }\alpha & \mathrm{for}\;\alpha \;\leq \;1 \end{array}$$

(A26) $$\alpha \;=\;\frac{{f^{\prime }}_{1,t}\;{f^{\prime }}_{2,t}}{{{f^{\prime }}_{12,v}}^{2}}$$

where

f′_1,t_, f′_2,t_ and f′_12,v_—strength of a virtually unidirectional lamina with the same fiber volume fraction V_f_ as the CSM layer.
